# Economic evaluations of eHealth technologies: A systematic review

**DOI:** 10.1371/journal.pone.0198112

**Published:** 2018-06-13

**Authors:** Chiranjeev Sanyal, Paul Stolee, Don Juzwishin, Don Husereau

**Affiliations:** 1 School of Public Health and Health Systems, University of Waterloo, Waterloo, Canada; 2 Health Technology Assessment and Innovation, Alberta Health Services, Edmonton, Alberta, Canada; 3 Institute of Health Economics, Edmonton, Alberta, Canada; University of Ottawa, CANADA

## Abstract

**Background:**

Innovations in eHealth technologies have the potential to help older adults live independently, maintain their quality of life, and to reduce their health system dependency and health care expenditure. The objective of this study was to systematically review and appraise the quality of cost-effectiveness or utility studies assessing eHealth technologies in study populations involving older adults.

**Methods:**

We systematically searched multiple databases (MEDLINE, EMBASE, CINAHL, NHS EED, and PsycINFO) for peer-reviewed studies published in English from 2000 to 2016 that examined cost-effectiveness (or utility) of eHealth technologies. The reporting quality of included studies was appraised using the Consolidated Health Economic Evaluation Reporting Standards statement.

**Results:**

Eleven full text articles met the inclusion criteria representing public and private health care systems. eHealth technologies evaluated by these studies includes computerized decision support system, a web-based physical activity intervention, internet-delivered cognitive behavioral therapy, telecare, and telehealth. Overall, the reporting quality of the studies included in the review was varied. Most studies demonstrated efficacy and cost-effectiveness of an intervention using a randomized control trial and statistical modeling, respectively. This review found limited information on the feasibility of adopting these technologies based on economic and organizational factors.

**Conclusions:**

This review identified few economic evaluations of eHealth technologies that included older adults. The quality of the current evidence is limited and further research is warranted to clearly demonstrate the long-term cost-effectiveness of eHealth technologies from the health care system and societal perspectives.

## Introduction

In developed countries including Canada, life expectancy among individuals 65 years and older has grown due to health technology innovations and advancements in public health among other factors [1, 2]. The population aged 60 and older worldwide is expected to grow from 901 million in 2015 to 1.4 billion in 2030 [1]. The rise of the aging population is associated with increased new cases of cancer, dementia, and mental illness among others [2]. Older adults aged 65–79 and 80+ years have on average 3 or 4 chronic conditions, respectively [2]. The management of new cases and existing conditions will require substantial health care resources [1].

Electronic health (eHealth) technologies utilize information and communication technologies to manage health, deliver care, and manage the health care system [3]. eHealth technologies can play a role in allowing seniors to live at home [3], while increasing the efficiency of the health care system. For example, they may replace face-to-face meetings with health care professionals and provide patient education, counseling services, access to data (and/or collection) and enable health information sharing. In addition, they can facilitate patient monitoring (and support), clinical examination, diagnosis and treatment [3]. These technologies can bring the health care provider and health system to the patient thereby enabling patient-oriented care.

Economic evaluations can inform health care decision makers about efficient allocation of scarce resources to improve health outcomes. Cost-effectiveness (CE) and cost-utility (CU) studies are usually used by decision makers for value proposition of a novel health technology [4]. CE and CU analyses quantify costs and consequences using health outcomes (e.g. life years saved or functional improvement) and quality-adjusted-life-years (QALYs), respectively [4]. In the comparison between two potential health technologies or other interventions, the incremental cost-effectiveness ratio (ICER) quantifies the difference in cost (ΔC) divided by the difference in their effect (ΔE) [4]. An intervention is considered cost-effective if the ICER (ΔC/ΔE) is less than a predetermined maximum amount (λ) the payer is willing to pay (WTP) for a gain in health outcome (i.e. ΔC/ΔE<λ) [4]. Health care decision makers most widely use the ICER to make decisions on adoption and reimbursement of health technologies [4]. The objective of this study was to systematically review and appraise the quality of CE or CU studies assessing eHealth technologies in study populations involving older adults. Moreover, we examined the literature to know whether any conclusion on CE or CU can be made on the use of eHealth technologies in older adults.

## Methods

### Literature search strategy

This systematic review is reported in accordance with the reporting guidance provided by the Preferred Reporting Items for Systematic Reviews and Meta-Analysis (PRISMA) statement [5]. An experienced health science librarian searched the literature published between January 1^st^, 2000 to October 4^th^, 2016 in the following electronic databases: Medline, EMBASE, CINAHL, NHS EED, and PsycINFO. Innovations in eHealth technologies have evolved considerably over the years; therefore, we decided to limit the literature search to articles published in 2000 or later. The search used text words 'assistive technology', 'socially assistive robots', 'mobile health', 'mobile robot', 'smart home system', 'telecare', 'telehealth', 'telemedicine', 'wander prevention systems', 'mobile locator devices', 'gps', 'location based technology', 'mobile apps', 'mobile application', 'cell phone', 'web based', 'internet', 'mhealth', 'm health', ‘eHealth’ or 'e health' cross referenced with 'older adult', 'elderly', 'seniors', or 'older patient' and 'cost effective', 'cost utility', or 'economic evaluation', S1 Text. The reference lists of the included studies were hand searched to identify additional publications.

### Selection of studies

This review focussed on studies that conforms to PICO (population, intervention, comparison, and outcomes) criteria older adults, eHealth technologies, standard or usual care and ICER, respectively [6, 7]. Therefore, this review focused on the ICER as the outcome of interest.

Two reviewers reviewed titles, abstracts, and full text articles. Titles and abstracts were screened for relevance based on the research question. Any articles that met all of the inclusion criteria were retained for full text review. The reviewers independently read full text of eligible articles. Disagreements were resolved by consensus between the two reviewers; where they did not reach consensus, this was adjudicated by a third reviewer.

Following inclusion/exclusion criteria were considered to identify relevant studies:

Inclusion criteria -
  1. Peer reviewed studies published in English.
  2. CE or CU studies of eHealth technologies that was conducted alongside a clinical trial or based on simulation modeling.
  3. Study population included older adults that is, individuals on average aged 60 years or older.
Exclusion criteria -
  1. Letters to the editor, conference abstracts, review articles without original data, or grey literature and/or reports.
  2. ICER not reported.
  3. Cost analysis studies (i.e. studies which measured or compared costs without health outcomes).

### Data abstraction

Data abstraction for each of the studies in the review included the following information: country, year of publication, intervention, comparator, disease, mean age, sample size, efficacy-effectiveness study design, CE or CU method, perspective, time horizon, year of costing, ICER, and funding source.

### Quality assessment

We used the Consolidated Health Economic Evaluation Reporting Standards (CHEERS) statement to appraise the quality of reporting of the studies [8]. The CHEERS statement includes 24 items that appraises an article on the following criteria: (i) title and abstract, (ii) introduction, (iii) methods, (iv) results, (v) discussion, and (vi) funding and conflict of interest [8].

## Results

### Overview of studies included

**Fig 1:**
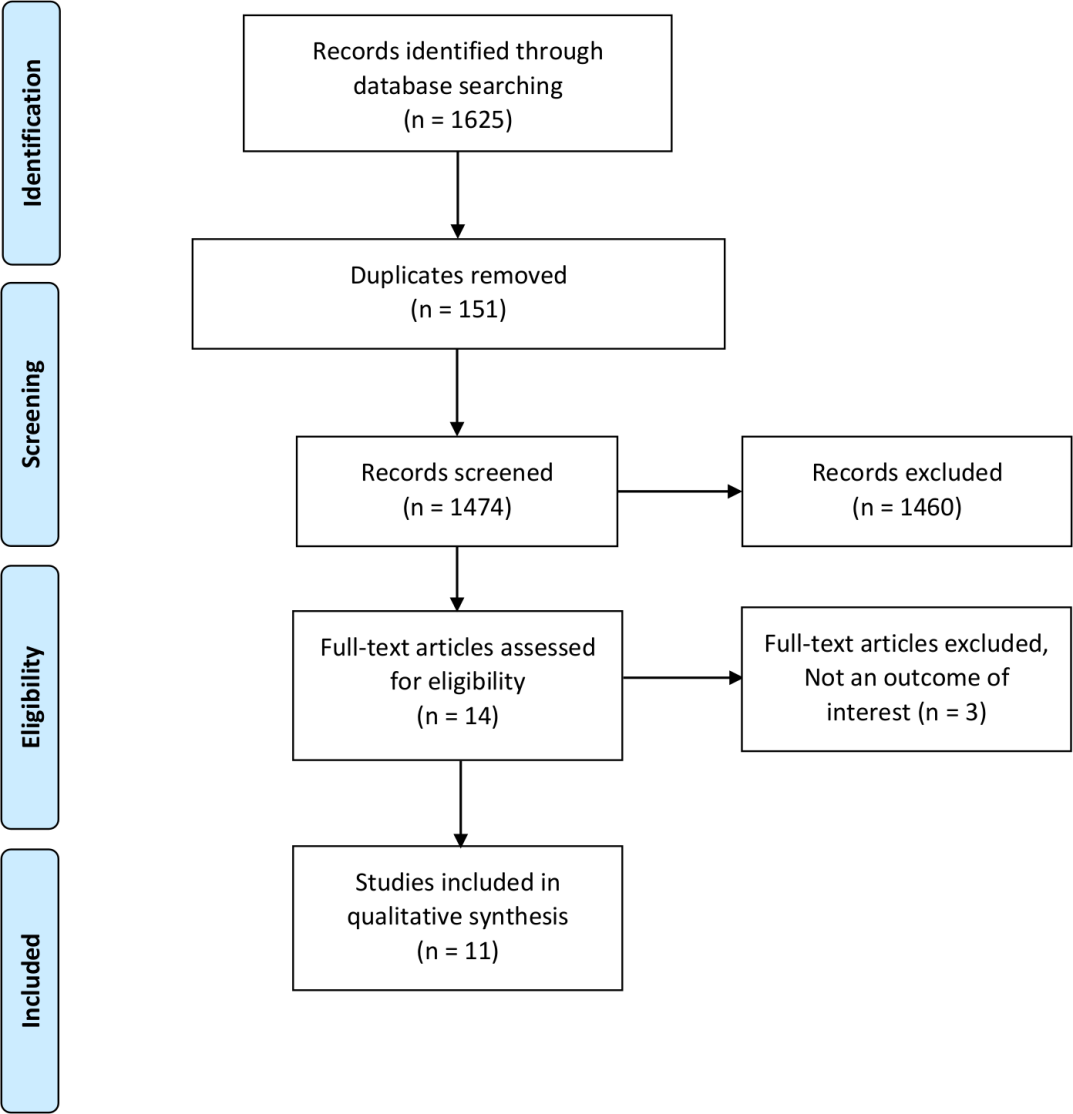
Study selection and identification flowchart.

Of the 1474 records identified after removing duplicates, 14 potentially relevant full text articles were reviewed for eligibility, of which 11 studies met our eligibility criteria and were included in the review (Fig 1). The effectiveness (or efficacy) data of two CE studies were based on observational studies and the rest were based on single randomized controlled trials, Table 1. The mean age range of the study population for intervention and control groups were 64.5 to 75.9 and 64.2 to 73.2 years, respectively, Table 1. The sample sizes in the intervention and control group were (n = 24 to 1699) and (n = 21 to 1692), respectively, Table 1.

**Table 1:** Characteristics of the included studies.

Author, country, year	Intervention vs. comparator	Disease	Mean age, years (SD)	Sample size	Efficacy-effectiveness study design	Modeling method	Perspective	Time horizon	Year of costing	ICER	Funding source
Barnett et al., USA, 2007 [9]	Care coordination/home telehealth (CCHT)	Chronic disease (e.g. diabetes)	68.2 (9.2)	370	Pre-post analysis	Regression analysis	NR	1-year	NR	$60,941 /QALY	Public
Cleveringa et al., The Netherlands, 2010 [13]	Diabetes care program (DCP) vs. Usual care (UC)	Diabetes	DCP-65.2 (11.3) UC-65.0 (11.0)	DCP—1699 UC—1692	RCT	Micro-simulation and regression analysis	Dutch health care	Lifetime	NR	€38,243 /QALY	Private
Boyne et al., The Netherlands, 2013 [14]	Telemonitoring (TM) vs. Usual care (UC)	Congestive heart failure	TM—71.0 (11.9) UC—71.9 (10.5)	TM—197 UC—185	RCT	Statistical method	Dutch health care	1-year	2008	€40,321 /QALY	Public and private
Cui et al., Canada, 2013 [15]	Standard care + Health Lines (HL) vs. Standard care + Health Lines + in house monitoring (HLM) vs. Standard care (SC)	Congestive heart failure	Overall—75 (12)	HL—61 HLM—58 SC—55	RCT	Regression analysis	Health care	1-year	2005	$2,975/QALY SC dominated by HL and HLM	NR
Peels et al., The Netherlands, 2014 [16]	Print delivered instructions (Print) vs. Web delivered instructions (Web) vs. Usual care (UC)	Metabolic equivalents of physical activity for chronic diseases	Print—63.1, 64.0 (8.7, 9.4) Web—61.8, 60.8 (7.1, 7.5) UC—64.2 (9.5)	Print—439, 435 Web—423, 432 UC—411	RCT	State transition simulation modeling	NR	Lifetime	2011	Print - €7,500/QALY Web - €10,100/QALY	Public
Henderson et al., UK, 2014 [17]	Telecare (TC) vs. Usual care (UC)	Chronic disease (e.g. diabetes, heart failure etc.)	65–74†	TC—1276 UC—1324	Pragmatic RCT	Regression analysis	National Health Service or local authorities	12-months	2009- 2010	£297,000/QALY	Public
Author, country, year	Intervention vs. comparator	Disease	Mean age, years (SD)	Sample size	Efficacy-effectiveness study design	Modeling method	Perspective	Time horizon	Year of costing	ICER	Funding source
Jo´dar-Sa´nchez et al., Spain, 2014 [10]	Telehealth (TH) vs. Control group (CG)	Chronic obstructive pulmonary disease	TH—74.4 (7.6) CG—70.8 (10.4)	TH—24 CG—21	RCT	Statistical method	National Health Service	4-months	2014	€223,726/QALY	Public
Dear et al., Australia, 2015 [18]	Internet delivered CBT (iCBT) vs. Waitlist control (WC)	Generalized anxiety disorder	iCBT—65.4 (4.7) WC—65.5 (5.8)	iCBT– 35 WC—37	RCT	Regression analysis	National health provider	12-months	NR	$8,806/QALY	Non government organization
Titov et al., Australia, 2015 [19]	Internet delivered CBT (iCBT) vs. Waitlist control (WC)	Depression	iCBT—64.5 (2.6) WC—66.2 (3.8)	iCBT—27 WC– 25	RCT	Regression analysis	National health provider	12-months	NR	$4,392/QALY	Non government organization
Dixon et al., UK, 2016 [11]	Healthlines service + usual care (HL) vs. Usual Care (UC)	Cardio vascular disease	Men—67 Women—69	HL-325 UC-316	Pragmatic RCT	Regression analysis	UK National Health Service	Lifetime	2012- 2013	£2,091/QALY	Public
Dixon et al., UK, 2016 [12]	Healthlines service + usual care (HL) vs. Usual Care (UC)	Cardio vascular disease	67.2	HL-325 UC-316	Pragmatic RCT	Cohort simulation model	UK National Health Service	12- months	2012- 2013	£10,859/QALY	Public

SD—standard deviation, RCT—randomized control trial, ICER–incremental cost effectiveness ratio, NR—not reported, QALY—quality adjusted life year, ^†^most common age group

eHealth technologies evaluated were telehealth [9–12], a computerized decision support system [13], telemonitoring [14, 15], web-based physical activity intervention [16], telecare [17], and internet delivered cognitive behavior therapy (iCBT) [18, 19] compared with usual (or standard) care in patients with diabetes [9, 13, 16, 17], congestive heart failure [14, 15, 17], cardiovascular disease [11, 12], colon cancer [16], breast cancer [16], acute myocardial infarctions [16], stroke [16], chronic obstructive pulmonary disease [10], generalized anxiety disorder [18], and depression [19]. These studies were conducted from the perspective of public health care system (i.e. The Netherlands [13, 16], Canada [15], United Kingdom [11, 12, 17], Spain [10], and Australia [18, 19]). Two studies used simulation modeling [11, 16], nine studies used statistical (regression) modeling [9, 10, 12, 14, 15, 17–19], and one used both simulation and regression modeling [13] to predict costs and QALYs. The time horizon of analysis were 4-months [10], 1-year [9, 12, 14, 15, 17–19], 5-years [16], 10-years [16], and lifetime [11, 13, 16]. The choice of time horizon used in the analysis was not justified by these studies. Most of the studies included in the review were funded by public agencies [9–12, 16, 17], followed by non-profit organisation [18, 19], private [13], and mixed (private and public) [14].

### Quality assessment of the included studies

**Fig 2:**
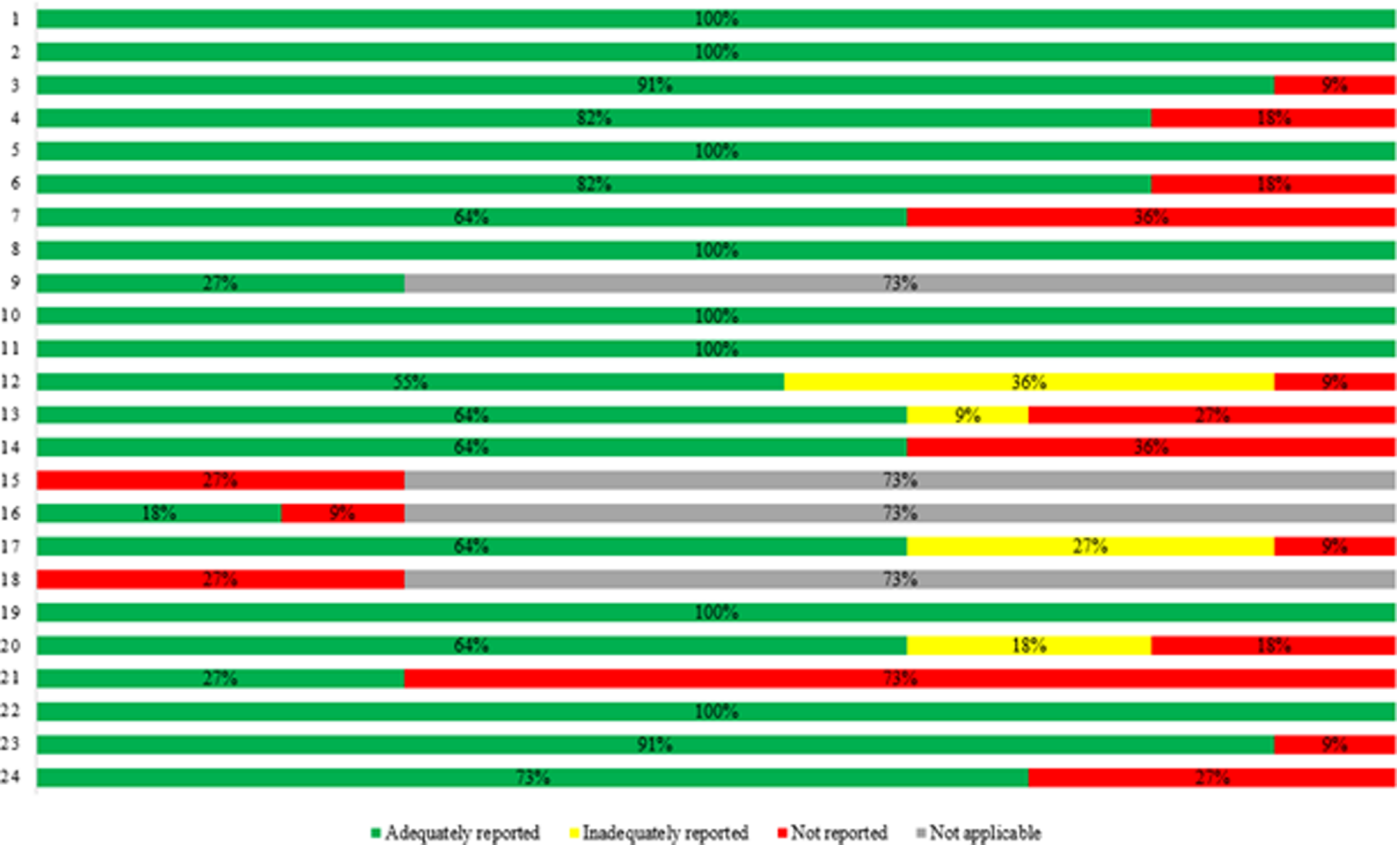
CHEERS statement quality results. Items: (1) Title, (2) Abstract, (3) Background and Objectives, (4) Target population and subgroups, (5) Setting and Location, (6) Study perspective, (7) Comparators, (8) Time horizon, (9) Discount rate, (10) Choice of health outcomes, (11) Effectiveness, (12) Preference valuation, (13) Estimate resources and costs, (14) Currency, price date, conversion, (15) Choice of model, (16) Assumptions, (17) Analytical methods, (18) Study parameters, (19) Incremental costs and outcomes, (20) Uncertainty—single study or model based, (21) Heterogeneity, (22) Study findings/limitations/generalizability/current knowledge, (23) Source of funding, (24) Conflict of interest.

The reporting quality of the studies is presented in Tables 2, S1 and Fig 2 which reflects the strengths and limitations of these studies. The major findings are summarized below.

**Table 2:** Quality assessment of included studies using CHEERS statement.

	Section/item	Percentage (%) of studies			
		Adequately reported	Inadequately reported	Not reported	Not applicable
**Title and abstract**	Title	100	-	-	-
	Abstract	100	-	-	-
**Introduction**	Background and Objectives	91	-	9	-
**Methods**	Target population and subgroups	82	18	-	-
	Setting and Location	100	-	-	-
	Study perspective	82	-	18	-
	Comparators	64	-	36	-
	Time horizon	100	-	-	-
	Discount rate	27	-	-	73
	Choice of health outcomes	100	-	-	-
	Effectiveness	100	-	-	-
	Preference valuation	55	36	9	-
	Estimate resources and costs	64	9	27	-
	Currency, price date, conversion	64	-	36	-
	Choice of model	-	-	27	73
	Assumptions	18	-	9	73
	Analytical methods	64	27	9	-
**Results**	Study parameters	-	-	27	73
	Incremental costs and outcomes	100	-	-	-
	Uncertainty—single study or model based	64	18	18	-
	Heterogeneity	27	-	73	-
**Discussion**	Study findings/limitations/generalizability/current knowledge	100	-	-	-
**Other**	Source of funding	91	-	9	-
	Conflict of interest	73	-	27	-

#### Study objective, population characteristics and comparators

The findings of a CE or CU study may vary based on the characteristics of the population included in a study. Most of the studies included in the review clearly stated their objectives and population characteristics. One study did not clearly state the study objective [16], two studies inadequately described study population characteristics [10, 17]. Four studies did not clearly describe the comparator (e.g. usual care) against which the intervention of interest was compared [10–12, 15].

#### Study perspective

The perspective of a study guides the cost components and outcomes to be evaluated by a study. Most of these considered the health care perspective; hence, the broader societal perspective was not represented. Two studies did not clearly state the perspective of the study [9, 16]. However, the reporting indicates these studies [9, 16] were most likely conducted from the health care system perspective.

#### Source of efficacy or effectiveness data

All included studies were based on single experimental [10–19] or non-experimental [9] study. The study design to evaluate the efficacy or effectiveness of these technologies was adequately described within the word limit of the journals. However, these studies did not clearly state why single efficacy or effectiveness study was used to understand whether the best available evidence was considered for the decision problem addressed by the study. Further, experimental designs comprised of randomized control trials (RCTs) [10, 13–16, 18, 19] and pragmatic trials [11, 12, 17]. Of note, in contrast to RCTs, pragmatic trials have greater external validity which better reflects real world scenarios.

#### Preference based health outcomes

All studies measured QALYs to quantity health outcomes pertaining to the intervention of interest and the comparator. Most studies [9–12, 14, 15, 17–19] reported the preference based instrument (e.g. SF-36 or EQ-5D) used to generate QALYs. In addition, few studies [11, 12, 14, 18, 19] have reported using country specific value sets to reflect the local context. These studies did not clearly state the preference elicitation methods used by the instruments (e.g. standard gamble or time trade off) to realize whether the instrument can adequately represent decision under uncertainty [4].

#### Measurement of resource use and costing

Few studies [10, 14–16, 18, 19] clearly described health care resource use and cost components considered to address their research question. The year of costing to address inflation was reported by most of the studies [10–12, 14–17]. These studies did not clearly state whether costing was based on top-down or micro-costing was not clearly stated by these studies [7]. The review identified selective reporting on health care resource use and cost components by the studies.

#### Methods for base case and uncertainty analysis

The included studies analyzed patient level data to estimate the ICER. Studies have used either regression [9, 10, 12, 14, 15, 17–19], simulation modeling [11, 16] or both [13]. These studies used various regression methods to address specific data requirement (e.g. clustering, correlation between the error terms and costs or QALYs etc.). Two studies [13, 14] reported the influence of patient subgroups on the ICER. These studies used bootstrapping to estimate 95% confidence interval to reflect the uncertainty associated with the estimate.

The model based studies [11, 13, 16] did not clearly describe the methods used to estimate model parameters, list of parameters and state transition probabilities used to develop the model. Schematic representation of the model indicating health state transitions considered and potential transition trajectories were not reported. Inadequate reporting was observed with regard to uncertainty analysis (deterministic or probabilistic) [11, 13, 16]. For probabilistic sensitivity analysis the parameter distributions, ranges etc. used to assess uncertainty was not reported [11, 13, 16]. The uncertainty with the data was reflected by 95% confidence interval. Cost effectiveness acceptability curves were reported to highlight the probability of the eHealth technology being CE at varying thresholds of willingness-to-pay [11, 16].

## Discussion

CE or CU studies are important to assess the value for money of novel health technologies and are widely used for the adoption and funding decisions by governments, stakeholders, insurers, and health policy makers [4, 7]. This study sought to systematically review and critically appraise the existing literature on CE or CU of eHealth technologies involving older adults. Eleven studies included in the review evaluated various eHealth technologies and comparators in the management of chronic diseases, using different outcomes. The CHEERS statement [8] helped assess the quality of reporting of these studies.

Most of the studies included in this review used the clinical evidence generated by a single RCT. Therefore, generalizability of findings is limited since RCT’s are controlled studies and may not represent routine clinical practice well. The majority of the studies lacked detailed description on unit costs, data sources, and cost calculations. The methodology used to calculate costs can significantly influence the overall cost estimates. It is possible the word limit of journals may prevent authors from providing detailed description of health services resource use, corresponding unit cost, and cost calculation. However, reporting these details can help decision makers understand what costs were considered for the analyses and the extent to which they are pertinent to their settings. Most of the studies used a time horizon of 1-year and failed to assess long-term costs and QALYs. Further, the rationale for the choice of time horizon for the analyses was not stated by these studies. The choice of time frame may influence study findings. Given that these studies were conducted in patients with chronic diseases, a longer time horizon will be preferred to adequately represent long term health services use, costs, and QALYs.

Studies identified in this review were conducted in various countries, in the context of those countries’ health care systems. Applying results from differing contexts is difficult due to variations in clinical practice, unit costs, health care delivery, and perspective of analysis, among others. Consistent across studies was a lack of reporting on the feasibility of adopting these technologies based on economic and organizational factors. For example, whether adopting these technologies will lead to an increase in health care spending or in resources for staff training and change management (among others) was not discussed. Such insight will help decision makers to decide upon various scenarios to consider while adopting and implementing the novel technology into their specific context.

There are limitations associated with this systematic review. *First*, peer-reviewed studies published in English were considered for this study. Therefore, studies published in other languages were excluded by our search strategy. *Second*, our eligibility criteria was met by eleven studies that evaluated a range of eHealth technologies in the management of chronic diseases. Therefore, we were not able to categorize eHealth technologies by the type of technology or disease management. Moreover, included studies were of varying methodological rigor making it difficult to compare study findings. *Third*, this review included CE or CU studies of eHealth technologies that included older adults. Therefore, inclusion of other age groups can potentially result in more articles. *Finally*, we did not review the grey literature.

In light of the limitations of the existing evidence, more research on the cost-effectiveness of eHealth technologies is warranted. Future studies can potentially consider the following: conform to reporting statement [8] to demonstrate methodological rigor, meta-analysis (data permitting) to synthesize the clinical evidence or use real world data, descriptions of valuation of health services use and corresponding unit costs and costing methodology, adequate characterization of uncertainty with study findings using deterministic or probabilistic analysis, analysis from different perspectives, and use of longer time horizons accounting for various pathways used in routine disease management [20]. In order to support healthy aging in place, it will also be highly relevant to evaluate the cost-effectiveness of using eHealth technologies to deliver integrated care in older adults affected by Alzheimer's disease, arthritis, and other chronic diseases. Future studies may extend their analysis beyond “single-point-in-time” [21] technology adoption decision making [20, 21] and address questions from various perspectives (stakeholder, patient, public, health system, payer and/or industry) based on the context [22], continuum of care and life cycle evaluation of a health technology [20].

## Conclusions

eHealth technologies can be used to provide resource efficient patient-oriented care. This review identified growing use of these technologies in the management of chronic diseases in study populations including older adults. Given the limitations of these studies, there is a lack of convincing evidence to conclude whether the use of eHealth technologies to deliver health care to older adults will demonstrate value at any acceptable level of investment. It is important to improve the methodological rigour and reporting of CE or CU studies so that decisions to use eHealth technologies are informed by convincing evidence of a value proposition. In addition, studies should evaluate the long-term clinical and cost-effectiveness of these technologies along the continuum of care from health care and/or societal perspectives.

## Supporting information

S1 Table PRISMA checklist. (DOC)

S1 Text Search strategy (Medline). (DOCX)
