# Supplementary material for: Economic evaluations of eHealth technologies: A systematic review
Source: PLoS One. 2018 Jun 13;13(6):e0198112. doi: 10.1371/journal.pone.0198112 (PMC5999277; doi:10.1371/journal.pone.0198112)
Supplement: S1 Text — (DOCX) [file pone.0198112.s002.docx]

**S2 Search Strategy (Medline)**

| #1 | (assistive technology or socially assistive robots or mobile health or mobile robot or smart home system or telecare or telehealth or telemedicine or wander prevention systems or mobile locator devices or gps or location based technolog* or mobile apps or mobile application* or cell phone* or web based or internet or mhealth or m health or ehealth or e health).mp. or mobile applications/ or cellular phones/ or therapy, computer assisted/ or internet/ |
| --- | --- |
| #2 | (older adult* or elderly or seniors or older patient*).mp. or exp aged/ |
| #3 | (cost benefit* or cost effective* or cost utility* or economic evaluation or cost analysis).mp. or exp "Costs and Cost Analysis"/ |
| #4 | (english language and yr="2000 -Current") |
| #5 | #1 AND #2 AND #3 AND #4 |
